# Immuno-PET Molecular Imaging of RANKL in Cancer

**DOI:** 10.3390/cancers13092166

**Published:** 2021-04-30

**Authors:** Jonatan Dewulf, Christel Vangestel, Yannick Verhoeven, Jorrit De Waele, Karen Zwaenepoel, Peter A. van Dam, Filipe Elvas, Tim Van den Wyngaert

**Affiliations:** 1 Molecular Imaging Center Antwerp (MICA), Integrated Personalized and Precision Oncology Network (IPPON), Faculty of Medicine and Health Sciences, University of Antwerp, Universiteitsplein 1, B-2610 Wilrijk, Belgium; jonatan.dewulf@uantwerpen.be (J.D.); christel.vangestel@uantwerpen.be (C.V.); filipe.elvas@uantwerpen.be (F.E.); 2 Nuclear Medicine, Antwerp University Hospital, Drie Eikenstraat 655, B-2650 Edegem, Belgium; 3 Center for Oncological Research (CORE), Integrated Personalized and Precision Oncology Network (IPPON), Faculty of Medicine and Health Sciences, University of Antwerp, Universiteitsplein 1, B-2610 Wilrijk, Belgium; Yannick.Verhoeven@uantwerpen.be (Y.V.); Jorrit.DeWaele@uantwerpen.be (J.D.W.); Karen.Zwaenepoel@uza.be (K.Z.); peter.vandam@uza.be (P.A.v.D.); 4 Laboratory of Pathological Anatomy, Antwerp University Hospital, Drie Eikenstraat 655, B-2650 Edegem, Belgium; 5 Multidisciplinary Oncologic Centre Antwerp (MOCA), Antwerp University Hospital, Drie Eikenstraat 655, B-2650 Edegem, Belgium

**Keywords:** RANKL, antibody, PET, tumor microenvironment

## Abstract

**Simple Summary:**

Receptor activator of the nuclear factor kappa B ligand (RANKL) has been recently identified as a target of interest in the tumor microenvironment (TME), specifically in facilitating an immunosuppressive environment and subsequent resistance to immunotherapy. However, assessment of RANKL in the TME remains difficult due to its heterogeneous nature and suboptimal sampling methods. In our study we developed an anti-RANKL immuno-PET tracer to non-invasively monitor RANKL expression in the TME and help to understand the RANK/RANKL pathway.

**Abstract:**

Purpose: The involvement of RANK/RANKL signaling in the tumor microenvironment (TME) in driving response or resistance to immunotherapy has only very recently been recognized. Current quantification methods of RANKL expression suffer from issues such as sensitivity, variability, and uncertainty on the spatial heterogeneity within the TME, resulting in conflicting reports on its reliability and limited use in clinical practice. Non-invasive molecular imaging using immuno-PET is a promising approach combining superior targeting specificity of monoclonal antibodies (mAb) and spatial, temporal and functional information of PET. Here, we evaluated radiolabeled anti-RANKL mAbs as a non-invasive biomarker of RANKL expression in the TME. Experimental design: Anti-human RANKL mAbs (AMG161 and AMG162) were radiolabeled with ^89^Zr using the bifunctional chelator DFO in high yield, purity and with intact binding affinity. After assessing the biodistribution in healthy CD-1 nude mice, [^89^Zr]Zr-DFO-AMG162 was selected for further evaluation in ME-180 (RANKL-transduced), UM-SCC-22B (RANKL-positive) and HCT-116 (RANKL-negative) human cancer xenografts to assess the feasibility of in vivo immuno-PET imaging of RANKL. Results: [^89^Zr]Zr-DFO-AMG162 was selected as the most promising tracer for further validation based on biodistribution experiments. We demonstrated specific accumulation of [^89^Zr]Zr-DFO-AMG162 in RANKL transduced ME-180 xenografts. In UM-SCC-22B xenograft models expressing physiological RANKL levels, [^89^Zr]Zr-DFO-AMG162 imaging detected significantly higher signal compared to control [^89^Zr]Zr-DFO-IgG2 and to RANKL negative HCT-116 xenografts. There was good visual agreement with tumor autoradiography and immunohistochemistry on adjacent slides, confirming these findings. Conclusions: [^89^Zr]Zr-DFO-AMG162 can detect heterogeneous RANKL expression in the TME of human cancer xenografts, supporting further translation of RANKL immuno-PET to evaluate tumor RANKL distribution in patients.

## 1. Introduction

The receptor activator of nuclear factor kappa B ligand (RANKL) and its receptor RANK are members of the tumor necrosis factor (TNF) and TNF receptor (TNFR) superfamily. The binding of RANKL to RANK results in the trimerization of the receptor and recruitment of TNF receptor-associated factors (TRAF), adaptor proteins, and activation of downstream signaling pathways [1]. The RANKL/RANK system was initially discovered during research into TNFR homologs expressed on T cells and dendritic cells [2]. Subsequently, the RANK/RANKL interaction’s pivotal role in osteoclastogenesis and bone homeostasis was elucidated [3]. RANKL binds osteoprotegerin (OPG) and the leucine-rich repeat-containing G-protein coupled receptor 4 (LGR4), resulting in an inhibition of downstream signaling and acting as a negative feedback mechanism preventing excess activation. These findings have led to the development of RANKL-targeting therapies, of which the fully human monoclonal antibody denosumab has been in clinical use for almost a decade. Indeed, denosumab has proven benefits for patients with osteoporosis, cancer-related bone disease, and other skeletal conditions [4,5,6].

More recently, RANK/RANKL signaling was also found to be an essential component in carcinogenesis, specifically in the maintenance of self-renewal of cancer stem cells and up-regulation of anti-apoptotic pathways, making the RANK/RANKL axis an attractive therapeutic target [7]. Moreover, inhibiting RANKL has been shown to improve the effectiveness of immune-checkpoint inhibitors (ICI) targeting CTLA-4 or PD(L)-1 in preclinical models of cancer [8,9]. While immunotherapy has revolutionized contemporary oncology, it is typically only beneficial in a small subset of patients. This limitation of ICIs has fueled interest in an improved understanding of the intricate interplay of cancer cells, immune cells and cytokines in the tumor microenvironment (TME) [10]. To this end, current methods to assess RANKL expression typically include serum assays or ex vivo immunohistochemistry (IHC) of tissue biopsies. However, these techniques are hampered by issues with sensitivity, variability, and spatial heterogeneity within the TME and negatively impact patient comfort when tissue sampling is required, if tissue sampling is even remotely possible [11,12]. Immuno-positron emission tomography (PET) can provide solutions to the problems above by combining the superior sensitivity of PET with the benefits of high targeting specificity of monoclonal antibodies, which can provide information on whole-body biomarker distribution or tumor target expression and act as a companion diagnostic in vivo in a non-invasive and longitudinal manner.

We aimed to develop a non-invasive imaging biomarker using PET to study the expression and spatial heterogeneity of tumor expressed RANKL in the TME. In particular, ^89^Zr-anti-RANKL radioimmunoconjugates were synthesized in a reproducible way, characterized for antigen affinity and optimal biodistribution, and validated as markers of RANKL expression and heterogeneity in the TME in human cancer xenograft models.

## 2. Materials and Methods

### 2.1. Bioconjugation and Radiolabeling of RANKL-Targeted Monoclonal Antibodies (RANKL mAbs)

The human RANKL-targeting human monoclonal antibodies (mAbs) AMG161 (IgG1; Amgen Inc., Thousand Oaks, CA, USA) and AMG162 (human antibody; IgG2; denosumab (XGEVA), Amgen Inc., Thousand Oaks, CA, USA), and control IgG2 isotype (Sigma-Aldrich Cat# I5404, RRID:AB_1163681, Saint Louis, MO, USA) were modified with the bifunctional chelator deferoxamine (DFO/p-SCN-Bn-DFO, Macrocyclics, Plano, TX, USA). Briefly, unconjugated mAbs were prepared at 3 mg/mL in phosphate-buffered saline 0.01 M, pH 7.4 (PBS), buffer-exchanged with PBS solution and pH adjusted to 8.8–9.2 with 0.1 M Na_2_CO_3_ (Sigma Aldrich, St. Louis, MO, USA). Next, a 5-fold molar excess DFO was added to the AMG161, AMG162 and isotype IgG2 solutions and the conjugation was allowed to proceed for 1 h (AMG162) and 2 h (AMG161) at 37 °C with gentle agitation. Subsequently, the reaction mixture was purified using a PD-10 size exclusion column (Cytiva, Marlborough, MA, USA) and buffer-exchanged with PBS to remove unconjugated DFO using a 2 mL Amicon centrifugal filter unit with a 50 kDa cutoff (Merck, Darmstadt, Germany). The concentration of the final DFO-AMG161, -AMG162 and -IgG2 conjugates was determined via UV-VIS absorbance at 280 nm using a spectrophotometer (Genesys 10S UV-VIS, Thermo Fisher Scientific, Waltham, MA, USA).

The loading efficiency of the DFO chelates in the final bioconjugates was determined by ESI mass spectrometer (Centre for Proteomics, University of Antwerp) using a Q-TOF2 instrument (Waters, Milford, MA, USA), as previously described [13].

For ^89^Zr labeling, 185 MBq of ^89^Zr-oxalate (BV cyclotron VU, Perkin Elmer, Waltham, MA, USA) was diluted to 0.2 mL with 1 M oxalic acid (Sigma Aldrich, St. Louis, MO, USA), neutralized with 2 M Na_2_CO_3_ (Sigma Aldrich, St. Louis, MO, USA) and added to chelex-treated HEPES buffer (0.5 M, pH 7.2, Sigma Aldrich, St. Louis, MO, USA). This was followed by the addition of 1 mg of DFO-AMG162 or DFO-IgG2, or 0.75 mg of DFO-AMG161, and volume adjustment to 2 mL with HEPES buffer. Radiolabeling was performed for 1 h at 37 °C, after which the radiolabeled antibodies were purified using a PD-10 desalting column and concentrated using Amicon centrifugal filter units (cutoff 50 kDa). The radiochemical yield and purity were evaluated by size exclusion chromatography-high performance liquid chromatography (SEC-HPLC, Cytiva, Marlborough, MA, USA, Superdex 200 increase 5/150, phosphate buffer 0.05 M pH 6.7, λ = 280 nm) and instant thin-layer chromatography (iTLC, Elysia, Angleur, Belgium glass microfiber chromatography paper impregnated with a silica gel (SG), 20 mM citric acid/acetonitrile (9:1, (*v*/*v*)). iTLC strips were cut in half, and the bottom and top parts were counted for radioactivity in an automatic gamma-counter (Wizard 2480, PerkinElmer, Waltham, MA, USA). Radiolabeled antibody remained at the origin (Rf [^89^Zr]Zr-DFO-Antibody = 0), while free ^89^Zr and ^89^Zr-DFO moved with the solvent front (Rf = 1). The concentration of the radiolabeled antibody in the final PBS formulation was calculated using a spectrophotometer (Genesys 10S UV-VIS, λ = 280 nm), which was used to calculate the specific activity.

The radiotracers’ stability was evaluated over seven days in vitro by incubation in the final formulation (PBS) at room temperature and in human plasma and mouse plasma at 37 °C. Samples were spotted on TLC strips, and subsequent iTLC analysis was performed as described above.

The immunoreactivity of DFO-AMG161 and DFO-AMG162 was determined by using a non-cell based binding assay, as previously described [14].

### 2.2. Mice

Immunodeficient CD-1 nude female mice (Charles River Laboratories, Wilmington, MA, USA, RRID:IMSR_CRL:086), age 5–7 weeks, weight 20–25 g, were kept under environmentally controlled conditions (12 h light/dark cycle, 20–24 °C and 40–70% relative humidity) in individually ventilated cages with food and water ad libitum. Experimental procedures and protocols were performed following European Directive 86/609/EEC Welfare and Treatment of Animals and were approved by the local ethical committee (2018-48, University of Antwerp, Belgium). Sample size was estimated by power analysis (α = 0.05, power 0.8) and animals were assigned to experimental groups using simple randomization.

### 2.3. Ex Vivo Biodistribution

Mice (*n* = 4) were injected intravenously (i.v.) in the lateral tail vein with [^89^Zr]Zr-DFO-AMG161 (14 ± 0.7 µg; ~3.3 MBq; *n* = 20) or [^89^Zr]Zr-DFO-AMG162 (14 ± 0.7 µg; ~2.2 MBq; *n* = 20) in 200 µL sterile saline (0.9% NaCl). On days 1, 2, 3, 4 and 7 after radiotracer injection, blood was collected via cardiac puncture, and the mice were euthanized via cervical dislocation. Subsequently, blood, heart, lungs, liver, spleen, pancreas, stomach, small intestine, large intestine, kidneys, bladder, urine, muscle, fat, bone, brain and skin were harvested, weighed, and the radioactivity was measured using an automatic gamma-counter (Wizard 2480, PerkinElmer, Waltham, MA, USA). Uptake levels of the radiotracers were expressed as a percentage of the injected dose per gram (% ID/g). The radiotracer demonstrating the most favorable biodistribution was selected for subsequent xenograft experiments.

### 2.4. Xenograft Tumor Models

The HCT-116 human colorectal carcinoma cell line (ATCC Cat# CCL-247, RRID:CVCL_0291), the UM-SCC-22B human head and neck squamous carcinoma cell line (Laboratory of Experimental Cancer Research, Ghent University Hospital, Ghent, Belgium, RRID:CVCL_7732), and the ME-180 human cervical squamous carcinoma cell line (ATCC Cat# HTB-33, RRID:CVCL_1401) were selected for xenograft transplantation. The ME-180 cell line was transduced with human RANKL (#LTV2504, G&P Biosciences, Santa Clara, CA, USA) and selected with 4 mg/mL G418/Geneticin (ant-gn-1, InvivoGen Europe, Toulouse, France) for 5 days and will be referred to as ME-180-RANKL in the manuscript from hereon.

The HCT-116 and ME-180-RANKL cell lines were cultured in McCoy’s 5A modified medium (Invitrogen), and the UM-SCC-22B cell line was cultured in Dulbecco’s Modified Eagle medium (Invitrogen), both media were supplemented with 10 (*v*/*v*%) fetal bovine serum (FBS) (Invitrogen), 2 mM L-glutamine (Invitrogen), and 1 (m/m%) penicillin and streptomycin (Invitrogen). Cells were incubated at 37 °C in humidified conditions with 5% CO_2_. Cells were passaged and harvested using a 0.05% trypsin/EDTA solution.

The transduction efficiency was estimated by assessing RANKL expression using flow cytometry on a BD FACSAria II (BD Biosciences, San Jose, CA, USA) cytometer with an APC-conjugated antibody (#347508, Biolegend, San Diego, CA, USA). Positivity was determined using the overton algorithm.

Low-passage UM-SCC-22B (5 × 10^6^), HCT-116 (5 × 10^6^) and ME-180-RANKL (8 × 10^6^) cells were harvested, suspended in 100 µL sterile PBS and subcutaneously injected into the right hindlimb of athymic female CD-1 nude mice (Charles River Laboratories RRID:IMSR_CRL:086). When the tumors became palpable, tumor growth was evaluated 3 times/week using caliper measurements. Tumor volume was calculated according to the formula 0.5 × (length × width^2^).

Animals were eligible for the experimental design in the imaging study when tumor volume reached 100 mm^3^. Tumor bearing animal in which tumor growth was halted or did not exceed 100 mm^3^ were excluded from the study.

### 2.5. PET Imaging

Tumor-bearing mice were injected i.v. with [^89^Zr]Zr-DFO-AMG162 (73 ± 1.5 µg; ~10.5 MBq/mouse; *n* = 4–5) or [^89^Zr]Zr-DFO-IgG2 isotype control (75 ± 1.5 µg in 200 µL sterile saline; ~11.7 MBq/mouse; *n* = 5) via lateral tail vein injection. A blocking study was performed to confirm the binding specificity of [^89^Zr]Zr-DFO-AMG162. For this, mice bearing ME-180-RANKL xenografts (*n* = 5) were i.v. injected via the tail vein with a 50× excess dose of native (unconjugated and non-radioactive) AMG162 (3.75 mg, 150 µL) one day prior to radiotracer injection. PET/Computed Tomography (CT) images were acquired at 72 h and 120 h post-injection (p.i). Before image acquisition, mice were anesthetized using isoflurane (5% for induction, 2% for maintenance). Static whole-body PET images were acquired over 25 to 35 min using an Inveon small-animal PET/CT scanner (Siemens Healthineers, Erlangen, Germany). Following each PET acquisition, a whole-body CT scan of 10 min was acquired to obtain anatomic information for segmentation. Throughout the entire PET/CT scanning procedure, the mice were maintained at constant body temperature using a heating pad, and breathing was continuously monitored.

For quantitative analysis, volumes of interest were manually drawn on the PET images using PMOD (PMOD, v 3.6; PMOD Technologies, RRID:SCR_016547) to delineate the tumors. For an absolute measure of tracer uptake, normalized images were scaled according to the percent injected dose (% ID/mL = tissue uptake [kBq/mL]/injected dose [kBq] × 100%). After the last PET/CT imaging acquisition, mice were sacrificed via cervical dislocation, and ex vivo biodistribution was performed as described earlier.

### 2.6. Autoradiography and Immunohistochemistry Analysis

After gamma-counting, the tumors were rapidly snap-frozen in tissue-Tek (OCT compound; VWR, Radnor, PA, USA), sectioned (100 µm) using cryostat (Leica Biosystems, CM1950, Wetzlar, Germany), and exposed overnight to phosphor screen plates (Fujifilm, Tokio, Japan). Exposed plates were imaged in a phosphor imager system (Typhoon FLA7000; GE Healthcare, Cytiva, Marlborough, MA, USA) to visualize regional tracer distribution as qualitative measure. Image analysis occurred via ImageJ v1.53 (RRID:SCR_003070) and underwent pseudo-coloring transformation.

Moreover, adjacent frozen tumor sections (10 µm) were taken at regular intervals across the entire tumor volume and used for histologic analysis of RANKL expression. Quantification of RANKL staining was performed by calculating the percentage of DAB-stained (brown) area across two non-sequential whole-tumor sections using the immunohistochemistry (IHC) profiler plugin for ImageJ v1.53 (RRID:SCR_003070), as previously described [15]. Three to five tumors were evaluated per tumor type. The mean percentage of positive stained area per tumor was used to calculate differences between groups. RANKL levels were correlated to the corresponding ex vivo radiotracer uptake in the tumor.

### 2.7. Statistical Analysis

The data are presented as mean % ± one standard deviation (SD). Graphs, two-tailed unpaired *t*-tests, Pearson correlation and linear regression analysis were performed with GraphPad Prism version 6.01 (RRID:SCR_002798). *p* value < 0.05 was considered as statistically significant.

## 3. Results

### 3.1. Bioconjugation, Characterization, and Radiolabeling Of AMG161 and AMG162

We have developed two radiolabeled human mAbs AMG161 (IgG1) and AMG162 (IgG2), both targeting human RANKL. Radioimmunoconjugates were generated via a random amine conjugation strategy, using a DFO macrocyclic chelator. DFO-modified RANKL targeting (AMG161 and AMG162) immunoconjugates were analyzed by ESI-QTOF2 mass spectrometry analysis, indicating that conjugations yielded between 0 and 3 DFO chelators bound to AMG161 and AMG162 (Figure S1A,B).

A binding assay was performed to confirm the conservation of the binding affinity to human RANKL of the DFO immunoconjugates. AMG161 (Kd~0.23 nM; 95% CI 0.18–0.27), DFO-AMG161 (Kd~0.26 nM; 95% CI 0.20–0.33), AMG162 (Kd~0.13 nM; 95% CI 0.076–0.18) and DFO-AMG162 (Kd~0.26 nM; 95% CI 0.041–0.17) showed similar binding affinity between native antibodies and their corresponding DFO immunoconjugates (Kd = equilibrium dissociation constant between antibody and antigen). Binding specificity towards human RANKL was confirmed using a non-specific human IgG1, which showed a complete lack of binding, and using OPG (Kd~0.63 nM; 95% CI 0.42–0.84) (Figure S2).

Radiolabeling of the DFO immunoconjugates with ^89^Zr resulted in high radiochemical yield ([^89^Zr]Zr-DFO-AMG161: 95%; [^89^Zr]Zr-DFO-AMG162: 78%, non-decay corrected), purity (>99%) and specific activity ([^89^Zr]Zr-DFO-AMG161: 240 MBq/mg; [^89^Zr]Zr-DFO-AMG162: 152 ± 22 MBq/mg). The isotype control, [^89^Zr]Zr-DFO-IgG2, showed a radiochemical yield of 95% (non-decay corrected), a purity of >99% and specific activity of 157 MBq/mg.

Both radiotracers remained stable in PBS, human and mouse plasma measured up to 7 days in vitro (Figure S3A,B).

### 3.2. Biodistribution of Both Radiotracers in Healthy CD-1 Nude Mice

**Figure 1:**
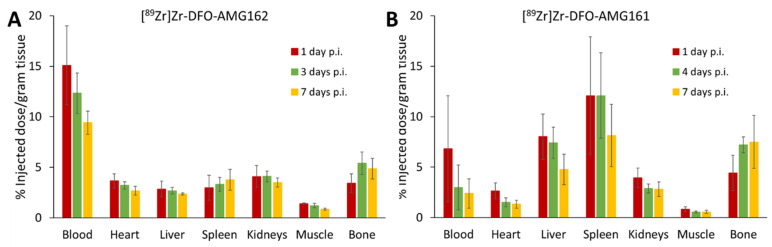
Ex vivo biodistribution of (**A**) [^89^Zr]Zr-DFO-AMG162 and (**B**) [^89^Zr]Zr-DFO-AMG161 in healthy CD-1 nude mice (*n* = 4) during 7 days post injection (p.i.). (Animals per timepoint: *n* = 4, data graph: mean +/− 1 standard deviation).

Differences in the biodistribution between the IgG1 and IgG2 isotype of the radiotracer were evaluated in vivo during 7 days in CD-1 nude mice. [^89^Zr]Zr-DFO-AMG162 showed an expected and slow antibody clearance from the blood, while undesirable non-specific radiotracer uptake in various organs was absent (Figure 1A, Table S1). In contrast, [^89^Zr]Zr-DFO-AMG161 demonstrated immediate high and variable sequestration in the liver and spleen in the majority of animals, clearing the antibody from circulation and undesirably reducing the exposure time of the radiotracer to a potential target (Figure 1B, Table S2). In light of these suboptimal findings, [^89^Zr]Zr-DFO-AMG161 was not further explored in subsequent xenograft experiments. On the other hand [^89^Zr]Zr-DFO-AMG162 showed favorable biodistribution and good characteristics as potential radiotracer.

### 3.3. [89. Zr]Zr-DFO-AMG162 PET Imaging Studies in ME-180-RANKL Transduced Subcutaneous Xenografts

The in vivo targeting potential of [^89^Zr]Zr-DFO-AMG162 was assessed in xenografts of the RANKL transduced ME-180 cell line. The ME-180-RANKL cell line was characterized using flow cytometry showing high RANKL expression (82.4% overton) compared to the non-transduced cell line (4.5% overton).

**Figure 2:**
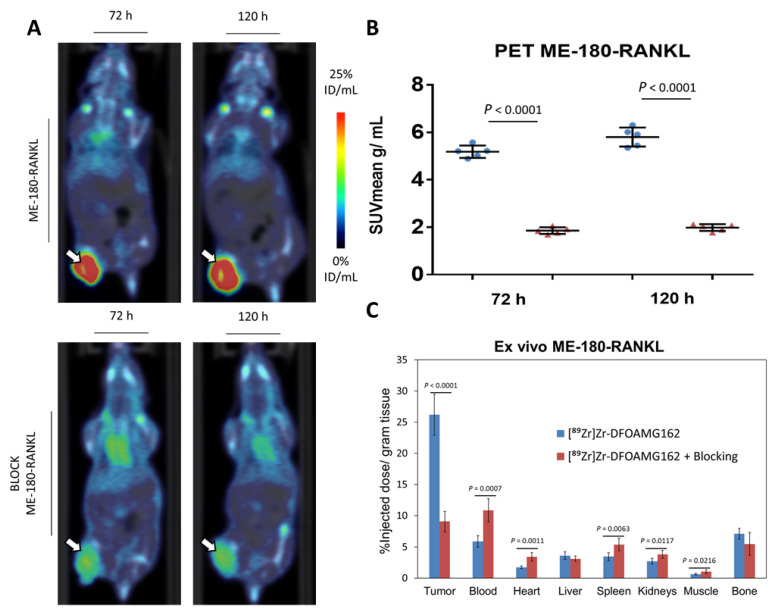
Overview imaging studies in ME-180-RANKL xenografts. (**A**) [^89^Zr]Zr-DFO-AMG162 PET imaging 72 h and 120 h post injection in ME-180-RANKL non-blocked (top) and blocked (bottom) xenografts (arrows). (**B**) Difference in tumor uptake (SUV_mean_ g/mL) assessed via PET between ME-180-RANKL xenografts treated without or with blocking dose at 72 h and 120 h p.i. (**C**) Ex vivo assessment of [^89^Zr]Zr-DFO-AMG162 biodistribution (% ID/g) at 120 h p.i. (Blue bars: [^89^Zr]Zr-DFO-AMG162 in non-blocked ME-180-RANKL xenografts, Red bars: [^89^Zr]Zr-DFO-AMG162 in blocked ME-180-RANKL xenografts, Animals per timepoint in both groups: *n* = 5, Data graph: mean +/− 1 standard deviation, White arrows: tumor, SUV_mean_: mean standard uptake value, ID/mL: injected dose/mL, Statistical analysis: unpaired student *t*-test).

[^89^Zr]Zr-DFO-AMG162 demonstrated a peak mean standardized uptake value (SUV_mean_) of 5.80 ± 0.40 g/mL at day 5, as shown in Figure 2A,B. The specificity of [^89^Zr]Zr-DFO-AMG162 was evaluated in a blocking study. Pre-injection of 50× excess native AMG162 significantly blocked radiotracer uptake in ME-180-RANKL tumors (1.98 ± 0.14 g/mL at day 5; *p* < 0.0001).

The ex vivo assessed biodistribution profile was measured at day 5 p.i. and showed a significantly higher radiotracer uptake (26.2 ± 3.3% ID/g; Figure 2C) in ME-180-RANKL xenografts when compared to blocked tumors (9.0 ± 1.6% ID/g; *p* < 0.0001). Significantly different radiotracer uptake between blocked and non-blocked xenografts could be observed in blood (*p* = 0.0007), heart (*p* = 0.0011), spleen (*p* = 0.0063), kidney (*p* = 0.0117) and muscle (*p* = 0.0216). The ex vivo biodistribution profile of [^89^Zr]Zr-DFO-AMG162 is summarized in Tables S3 and S4.

High tumor-to-organ ratios for [^89^Zr]Zr-DFO-AMG162 were observed in non-blocked ME-180-RANKL xenografts compared to the same xenografts with co-administration of a blocking dose Table 1.

High correlation was observed between PET and ex vivo biodistribution measurements in the tumor (*r* = 0.99, *p* < 0.0001). These results indicate that [^89^Zr]Zr-DFO-AMG162 enables non-invasive and specific detection of RANKL in vivo.

### 3.4. [89. Zr]Zr-DFO-AMG162 Imaging Studies in Human Head and Neck Squamous UM-SCC-22B Subcutaneous Xenografts

**Figure 3:**
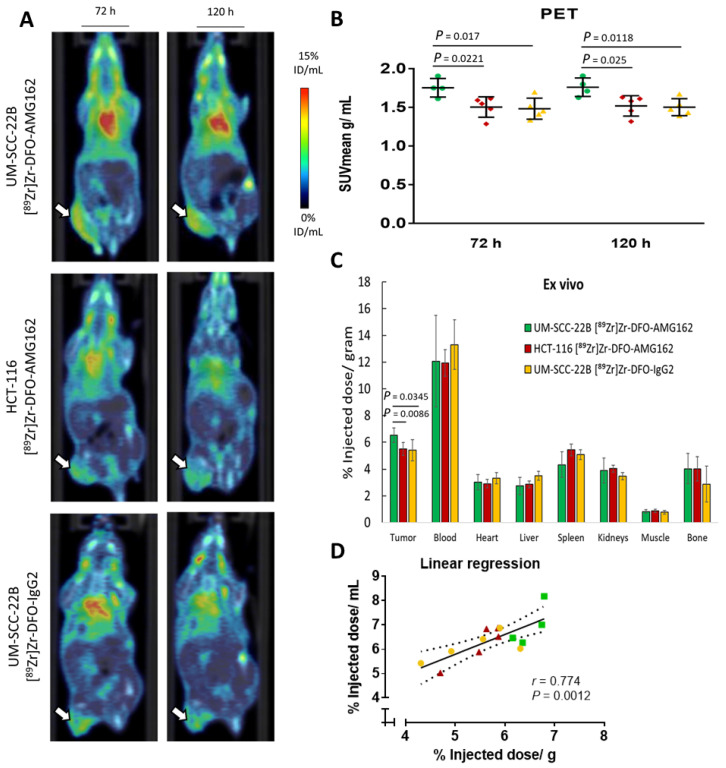
Overview imaging studies in UM-SCC-22B xenografts. (**A**) [^89^Zr]Zr-DFO-AMG162 PET imaging in UM-SCC-22B (top) and HCT-116 xenografts (middle), and [^89^Zr]Zr-DFO-IgG2 in UM-SCC-22B (bottom) at 72 h and 120 h p.i. (**B**) Difference in tumor uptake (SUV_mean_ g/mL) assessed via PET between UM-SCC-22B and HCT-116 xenografts imaged with [^89^Zr]Zr-DFO-AMG162 or [^89^Zr]Zr-DFO-IgG2 at 72 h and 120 h p.i. (**C**) Difference in tumor uptake as assessed ex vivo (% ID/g) between [^89^Zr]Zr-DFO-AMG162 and [^89^Zr]Zr-DFO-IgG2 in UM-SCC-22B and HCT116, and UM-SCC-22B xenografts, respectively. (**D**) Linear regression in UM-SCC-22B and HCT-116 xenografts between in vivo PET imaging and ex vivo measurements of [^89^Zr]Zr-DFO-AMG162 or [^89^Zr]Zr-DFO-IgG2 (*r* = 0.774, *p* = 0.0012). (Green bars: [^89^Zr]Zr-DFO-AMG162 in UM-SCC-22B xenografts, Red bars: [^89^Zr]Zr-DFO-AMG162 in HCT-116 xenografts, Yellow bars: [^89^Zr]Zr-DFO-IgG2 in UM-SCC-22B xenografts, Animals per timepoint in groups: *n* = 4–5, Data graph: mean +/− 1 standard deviation, White arrows: tumor, SUV_mean_: mean standard uptake value, ID/mL: injected dose/mL, Statistical analysis: unpaired student *t*-test).

The final goal of this study was to assess the ability of [^89^Zr]Zr-DFO-AMG162 to image physiological RANKL expression with respect to expression levels and distribution to support further clinical translation. [^89^Zr]Zr-DFO-AMG162 was injected in mice bearing UM-SCC-22B human head and neck squamous cell carcinoma (positive RANKL) and HCT-116 human colorectal cancer xenografts (negative RANKL control) tumors and PET/CT images were acquired at 3 and 5 days p.i. As non-targeted control, UM-SCC-22B xenografts were injected with [^89^Zr]Zr-DFO-IgG2 (non-specific human IgG2 isotype). PET/CT images at 5 days p.i. showed that [^89^Zr]Zr-DFO-AMG162 uptake in the tumors at day 5 was significantly higher in UM-SCC-22B xenografts (SUV_mean_ 1.76 ± 0.12 g/mL, *p* = 0.025) when compared with HCT-116 xenografts (SUV_mean_ 1.52 ± 0.13 g/mL) (Figure 3A,B). In addition, at day 5 we observed a significantly higher [^89^Zr]Zr-DFO-AMG162 SUV_mean_ (1.76 ± 0.12 g/mL, *p* = 0.0118) when compared with [^89^Zr]Zr-DFO-IgG2 (1.50 ± 0.11 g/mL) in UM-SCC-22B tumors, which confirmed selective [^89^Zr]Zr-DFO-AMG162 uptake beyond what can be explained by the enhanced permeability and retention (EPR) effect (Figure 3A,B). Radiotracer accumulation in other tissues was consistent with what was observed in the biodistribution experiments using healthy CD-1 nude mice.

To corroborate the PET results, ex vivo biodistribution analysis was performed at day 5 p.i., which confirmed significantly higher [^89^Zr]Zr-DFO-AMG162 uptake in UM-SCC-22B xenografts (6.5 ± 0.3% ID/g) compared with HCT-116 xenografts (5.5 ± 0.5% ID/g, *p* = 0.0086), and when compared with isotype control radiotracer uptake (5.4 ± 0.8% ID/g, *p* = 0.0345) (Figure 3C).

Tumor-to-organ ratios for different xenografts are shown in Table 2, showing the best ratios for [^89^Zr]Zr-DFO-AMG162 in RANKL positive UM-SCC-22B xenografts (Tables S5–S7).

A high correlation between in vivo radiotracer uptake (% ID/mL) and ex vivo measurement (% ID/g) in UM-SCC-22B xenografts (for [^89^Zr]Zr-DFO-AMG162 and [^89^Zr]Zr-DFO-IgG2) and in HCT-116 xenografts (for [^89^Zr]Zr-DFO-AMG162) was observed (*r* = 0.774, *p* = 0.0012; Figure 3D). Taken together these data show specific binding of [^89^Zr]Zr-DFO-AMG162 to RANKL in human head and neck squamous cancer xenografts UM-SCC-22B, compared to isotype control and a RANKL-negative xenograft.

### 3.5. Validation of the Radiotracer Uptake in Tumor Xenografts

**Figure 4:**
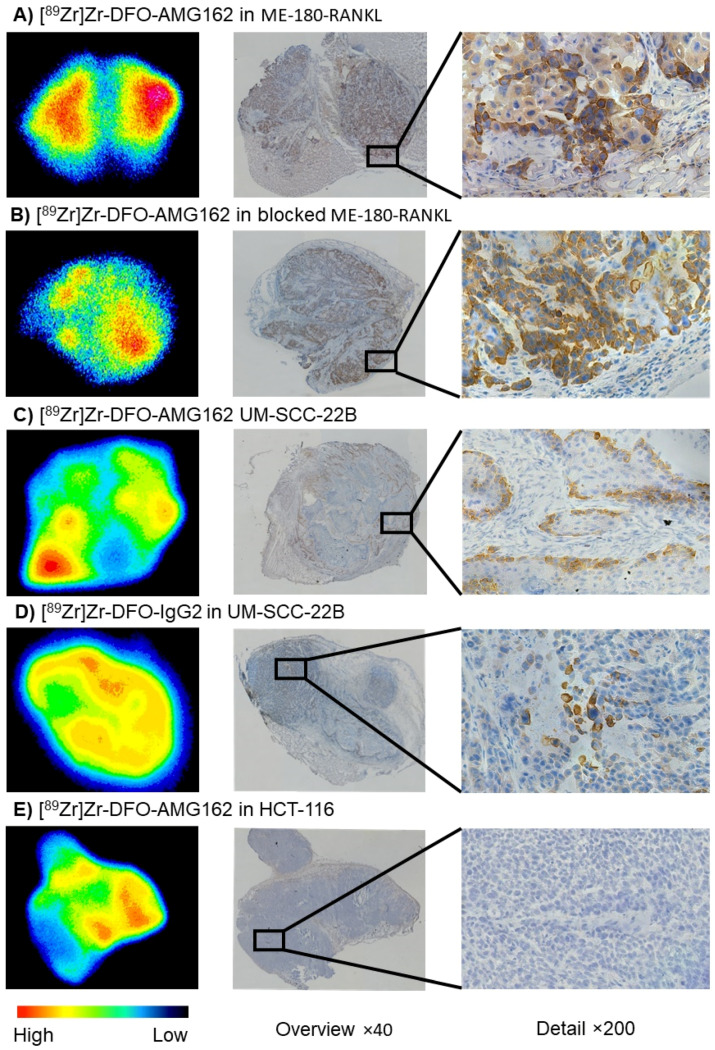
Autoradiography (High-Low (counts)), adjacent RANKL IHC (brown stain) overview image (×40), and detailed RANKL IHC image (×200) in xenografts: (**A**) [^89^Zr]Zr-DFO-AMG162 in ME-180-RANKL, (**B**) [^89^Zr]Zr-DFO-AMG162 in blocked ME-180-RANKL, (**C**) [^89^Zr]Zr-DFO-AMG162 in UM-SCC-22B, (**D**) [^89^Zr]Zr-DFO-IgG2 in UM-SCC-22B and (**E**) [^89^Zr]Zr-DFO-AMG162 in HCT-116.

Autoradiography (ARG) and IHC were performed in each group of animals, and the patterns of radiotracer distribution seen on ARG were compared with IHC staining on adjacent tumor sections. ARG of ME-180-RANKL tumor sections with [^89^Zr]Zr-DFO-AMG162 showed hot spots that could be matched entirely with IHC RANKL stainings (mean 24.0 ± 6.9% positively stained tumor area) in adjacent slides (Figure 4A). In the blocking experiments, fewer hot areas could be observed on ARG of ME-180-RANKL tumors, as expected (Figure 4B). However, these regions still demonstrated overlap with RANKL IHC stain (mean 23.8 ± 4.4% positively stained tumor area) (Figure S4).

Overall, a lower intensity of uptake was seen on ARG in UM-SCC-22B tumor sections evaluated with [^89^Zr]Zr-DFO-AMG162 (Figure 4C), but with good spatial congruency with RANKL expression on IHC. In contrast, no overlap between both methods was observed with [^89^Zr]Zr-DFO-IgG2 uptake (Figure 4D). Quantification of RANKL IHC of UM-SCC-22B xenografts with [^89^Zr]Zr-DFO-AMG162 and [^89^Zr]Zr-DFO-IgG2 showed a mean of 5.2 ± 3.1% and 5.0 ± 2.0% positively stained tumor area, respectively. Notably, UM-SCC-22B xenografts expressed high spatial RANKL heterogeneity. HCT-116 IHC slides were completely negative for RANKL stain, with a mean of 0.5 ± 0.2% positively stained tumor area (Figure 4E). In both UM-SCC-22B ([^89^Zr]Zr-DFO-IgG2, Figure 4D) and HCT-116 ([^89^Zr]Zr-DFO-AMG162, Figure 4E), radiotracer uptake corresponded mostly with regions of high vasculature and stroma, which is consistent with non-specific EPR uptake (Figure S4). Correlation analysis between RANKL IHC mean % positive stained tumor area and ex vivo or PET [^89^Zr]Zr-DFO-AMG162 uptake in all xenografts (blocking excluded) showed a correlation of *r* = 0.8634 (*p* < 0.0001) and *r* = 0.8934 (*p* < 0.0001), respectively.

## 4. Discussion

**Figure 5:**
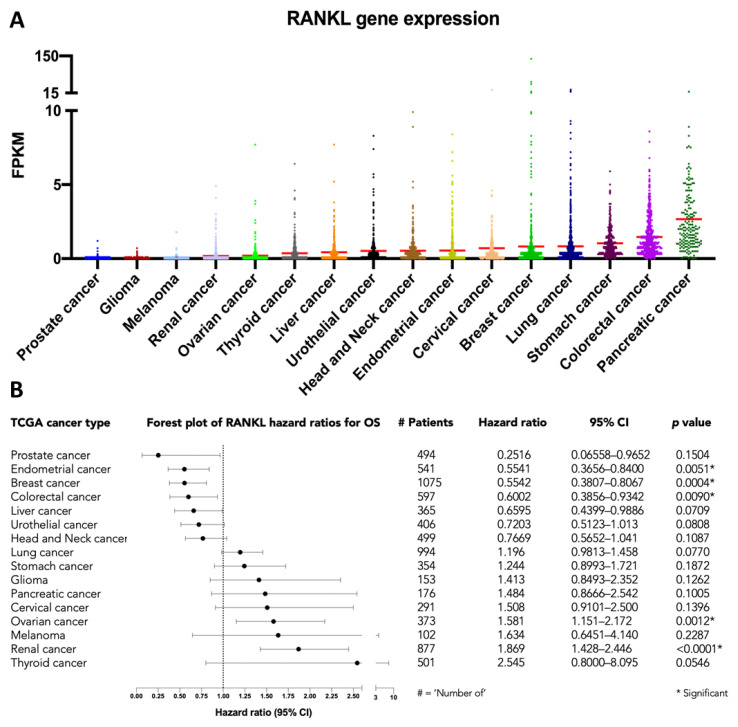
RANKL mRNA expression in 7798 patients with 16 different cancer types, available from The Cancer Genome Atlas (TCGA) database: (**A**) RANKL gene expression in Fragments Per Kilobase Million (FPKM), with red lines representing the mean, and (**B**) forest plot of the hazard ratios for overall survival (OS). Hazard ratios and the 95% confidence interval (CI) were estimated using the logrank method, as implemented in GraphPad.

The introduction of a RANKL-targeting treatment using the monoclonal antibody denosumab (AMG162) has improved clinical outcomes in patients with various skeletal conditions, including osteoporosis, metastatic bone disease, multiple myeloma, and giant cell tumor of bone [16]. Moreover, data from the Cancer Genome Atlas (TCGA) suggests that RANKL gene expression is associated with patient outcome in multiple cancer types in an explorative analysis of survival (Figure 5) [17,18]. This illustrates the potential of anti-RANKL therapies and novel methods for RANKL quantification, including immuno-PET.

More recently, the potential for repurposing denosumab as a modulator of the immune response in improving the efficacy of ICI in cancer treatment is actively being explored [19,20]. A phase II study in breast cancer patients supports this approach by showing an increase in lymphocytes and CD8+ T-cells in tumors exposed to single-agent denosumab (D-BEYOND; ClinicalTrials.gov Identifier: NCT01864798) [21]. Of note are two ongoing randomized trials, one being the CHARLI trial (ClinicalTrials.gov Identifier: NCT03161756) that is a phase Ib/II study including patients with unresectable stage III/IV melanoma treated with nivolumab in combination with four doses of denosumab, with or without ipilimumab (primary end-point: progression-free survival). The POPCORN trial (ACTRN12618001121257) is a phase Ib/II translational study including patients with stage IA to IIIA non-small-cell lung cancer receiving neoadjuvant treatment with two doses of nivolumab with or without denosumab (following nivolumab), followed by surgery and as primary end-point translational research into the tumor-immune correlates of combination therapy. Similar translational efforts are ongoing in phase I/II studies in cervical cancer (DICER; ISS20177041) and melanoma (ClinicalTrials.gov Identifier: NCT03620019) [1].

However, both in clinical practice and in the setting of clinical trials, questions remain regarding patient selection, optimal duration, long-term safety, maintenance dose, and sequencing of therapies that include a RANKL inhibitor [22,23]. These issues remain largely unaddressed, in part because of a lack of reliable non-invasive biomarkers for RANKL. Moreover, the observation that the benefit of the combination of RANKL and ICI may be sequence-dependent supports the concept of a biomarker that allows sequential assessment without the need for invasive procedures [24]. We initiated the search for an imaging biomarker of RANKL in the TME by radiolabeling AMG161 (IgG1) and AMG162 (IgG2). The difference in isotype showed no impact on in vitro characterization, with both yielding good radiolabeling, stability and unchanged affinity. However, a significant difference could be observed in vivo between both isotypes: [^89^Zr]Zr-DFO-AMG161 expressed different levels of radiotracer sequestration in spleen and liver, a phenomenon which could be related to mouse Fc receptor binding. Generally, IgG1 antibodies are more immunoreactive than their IgG2 counterparts and more prone to bind Fc receptors, an effect that is even more pronounced in immunodeficient mice models [25]. Even though the translational significance of this sequestration of the radiotracer for the biodistribution in humans is uncertain, it is an undesirable characteristic that can result in lower target uptake and more rapid metabolization of the tracer. Radiotracers revealed clear (5–10% ID/g) bone uptake, a phenomenon related to instability of the DFO complex, remarkably in patients the unwanted bone uptake is hardly an issue [26]. New chelators with improved characteristics have been developed (DFO*/HOPO) but were not yet commercially available at the start of this study [27]. Currently, AMG162 is an FDA-approved biopharmaceutical used in clinical care, whereas AMG161 is not, and this may limit its potential for successful clinical translation. For these reasons, [^89^Zr]Zr-DFO-AMG162 was selected for further experiments.

The selective uptake of [^89^Zr]Zr-DFO-AMG162 was demonstrated in RANKL-transduced ME-180 xenografts, with clear visualization on PET and high radiotracer uptake in the xenografts. Subsequent ARG and IHC showed corresponding regions with radiotracer uptake and staining, respectively. Moreover, a control blocking experiment reduced the radiotracer uptake demonstrating the specific uptake of the radiotracer.

Subsequent experiments were performed on patient-derived cell lines, UM-SCC-22B (RANKL positive) and HCT-116 (negative control). The RANKL positive cell line UM-SCC-22B was of interest since both membranes bound and soluble RANKL expression was reported. However, prior to the start of the study RNA RANKL expression was not assessed in different cell lines to select the highest RANKL expression cell line and is a limitation of the study.

[^89^Zr]Zr-DFO-AMG162 showed specific uptake of on immuno-PET in UM-SCC-22B xenografts and not of the radiolabeled isotype control [^89^Zr]Zr-DFO-IgG2. While we could visualize physiologically relevant amounts of tumor derived RANKL in the TME with [^89^Zr]Zr-DFO-AMG162, the RANKL expression of UM-SCC-22B was substantially lower compared to the transduced ME-180-RANKL cell line. In contrast, [^89^Zr]Zr-DFO-AMG162 uptake was significantly lower in HCT-116 xenografts (a non-RANKL expressing model) compared to UM-SCC-22B xenografts (a RANKL expressing cell line), supporting the specificity of tracer uptake. While the overall differences in mean SUV of [^89^Zr]Zr-DFO-AMG162 uptake between UM-SCC-22B and HCT-116 xenografts or over isotype control were low, remarkable differences in regional uptake were evident, confirming the considerable heterogeneity of RANKL in the TME, which may be missed by other sampling techniques (IHC). Autoradiography and immunohistochemistry were used to explore the contribution of RANKL heterogeneity on overall uptake. This showed convincing spatial congruency of [^89^Zr]Zr-DFO-AMG162 and RANKL expression in UM-SCC-22B xenografts. In contrast, autoradiography only demonstrated limited overlap in areas with high vasculature or stroma when using [^89^Zr]Zr-DFO-IgG2 (in UM-SCC-22B xenografts) or [^89^Zr]Zr-DFO-AMG162 (in HCT-116 xenografts), suggesting non-specific uptake. Indeed, the large size of antibodies (~150 kDa) represents an intrinsic limitation for efficient tumor diffusion, and due to their high avidity, they remain close to the periphery of the vasculature [28]. In addition, the long-blood half-lives of several days to weeks allows antibody-based radiotracers to achieve high uptake-values in targeted tissues, but simultaneously leads to elevated overall non-specific accumulation [29].Taken together, these data illustrate the potential benefits of immuno-PET for in vivo RANKL assessment compared to other sampling techniques, such as biopsies that are prone to error due to sampling bias or serum assays that are only able to provide a global biomarker quantification. In addition, immuno-PET has the unique capability of non-invasively visualizing actual drug-delivery to the TME.

Importantly, it is noted that while murine RANKL shares 83% sequence homology with human RANKL, the anti-RANKL antibodies used in our experiments have no affinity for murine RANKL [30]. For the purpose of our study, this difference is not of importance, but evidence does implicate the host-derived RANKL in the TME [31]. Therefore, further preclinical work using [^89^Zr]Zr-DFO-AMG162 to elucidate the tumor and host interactions in the TME will require more translationally appropriate clinical models. For example, the use of transgenic models and humanized mice models may better reflect the human TME and recapitulate the complex interactions of RANKL between the tumor and surrounding cells [32,33]. Finally, a more human-like TME may impact the levels of tumor-derived RANKL expression, resulting in improved imaging characteristics.

## 5. Conclusions

In conclusion, we describe for the first time in vivo assessment of RANKL expression in the TME using immuno-PET imaging. Our results suggest that RANKL imaging offers advantages over more traditional approaches for longitudinal tumor characterization and merits further investigation. [^89^Zr]Zr-DFO-AMG162 showed favorable stability, high binding affinity, and specific tumor uptake with very good visual agreement to the spatial distribution of RANKL in the TME as assessed with histology, supporting further translation to evaluate tumor RANKL distribution in patients.

## Figures and Tables

**Table 1:** Tumor-to-organ ratio of [^89^Zr]Zr-DFO-AMG162 5 days post injection in mice bearing ME-180-RANKL blocked (*n* = 5) and non-blocked (*n* = 5) xenografts. Data is shown below as mean ± the standard deviation.

Tumor/Organ Ratio	Blood	Muscle	Bone
ME-180-RANKL	4.5 ± 0.7	43.3 ± 4.6	3.7 ± 0.4
ME-180-RANKL + Blocking	0.8 ± 0.1	9.3 ± 3.1	1.8 ± 0.4

**Table 2:** Tumor-to-organ ratio 5 days post injection in mice bearing UM-SCC-22B ([^89^Zr]Zr-DFO-AMG162 (*n* = 4) and [^89^Zr]Zr-DFO-IgG2 (*n* = 5) and HCT-116 ([^89^Zr]Zr-DFO-AMG162 (*n* = 5) xenografts. Data is shown below as mean ± the standard deviation.

Tumor-to-Organ Ratio	Blood	Muscle	Bone
UM-SCC-22B ^RANKL+^ [^89^Zr]Zr-DFO-AMG162	0.61 ± 0.1	8.04 ± 1.4	1.74 ± 0.5
UM-SCC-22B ^RANKL+^ [^89^Zr]Zr-DFO-IgG2	0.41 ± 0.1	7.12 ± 1.2	2.27 ± 1.2
HCT-116 ^RANKL-^ [^89^Zr]Zr-DFO-AMG162	0.46 ± 0.03	6.53 ± 1.4	1.42 ± 0.3

## Data Availability

Data is contained within the article or supplementary material.

## Supplementary Materials

The following are available online at https://www.mdpi.com/article/10.3390/cancers13092166/s1, Figure S1: Mass spectrometry bioconjugates, Figure S2: Ligand assay, Figure S3: Stability of radio-immunoconjugates in vitro, Figure S4: IHC quantification, Table S1: Ex vivo biodistribution (mean ±1 standard deviation % ID/g) in healthy CD-1 nude mice (*n* = 4) at 1, 2, 3, 4, 7 days post injection of [^89^Zr]Zr-DFO-AMG162, Table S2: Ex vivo biodistribution (mean ±1 standard deviation % ID/g) in healthy CD-1 nude mice (*n* = 4) at 1, 2, 3, 4, 7 days post injection of [^89^Zr]Zr-DFO-AMG161, Table S3: Ex vivo biodistribution (mean ±1 standard deviation % ID/g) and tumor/organ ratio in healthy CD-1 nude mice (*n* = 5) at 5 days post injection of [^89^Zr]Zr-DFO-AMG162 in ME-180-RANKL xenografts, Table S4: Ex vivo biodistribution (mean ±1 standard deviation % ID/g) and tumor/organ ratio in healthy CD-1 nude mice (*n* = 5) at 5 days post injection of [^89^Zr]Zr-DFO-AMG162 blocked ME-180-RANKL xenografts, Table S5: Ex vivo biodistribution (mean ±1 standard deviation % ID/g) and tumor/organ ratio in healthy CD-1 nude mice (*n* = 4) at 5 days post injection [^89^Zr]Zr-DFO-AMG162 in UM-SCC-22B xenografts, Table S6: Ex vivo biodistribution (mean ±1 standard deviation % ID/g) and tumor/organ ratio in healthy CD-1 nude mice (*n* = 5) at 5 days post injection [^89^Zr]Zr-DFO-IgG2 in UM-SCC-22B xenografts, Table S7: Ex vivo biodistribution (mean ±1 standard deviation % ID/g) and tumor/organ ratio in healthy CD-1 nude mice (*n* = 5) at 5 days post injection [^89^Zr]Zr-DFO-AMG162 in HCT-116 xenografts.
