# Supplementary material for: Immuno-PET Molecular Imaging of RANKL in Cancer
Source: Cancers (Basel). 2021 Apr 30;13(9):2166. doi: 10.3390/cancers13092166 (PMC8124205; doi:10.3390/cancers13092166)
Supplement: Supplementary file 1 [file cancers-13-02166-s001.zip › cancers-1173573-supplementary.pdf]

Supplementary Materials: Immuno-PET Molecular Imaging of RANKL in Cancer

Jonatan Dewulf, Christel Vangestel, Yannick Verhoeven, Jorrit De Waele, Karen Zwaenepoel, Peter A. van Dam, Filipe Elvas and Tim Van den Wyngaert

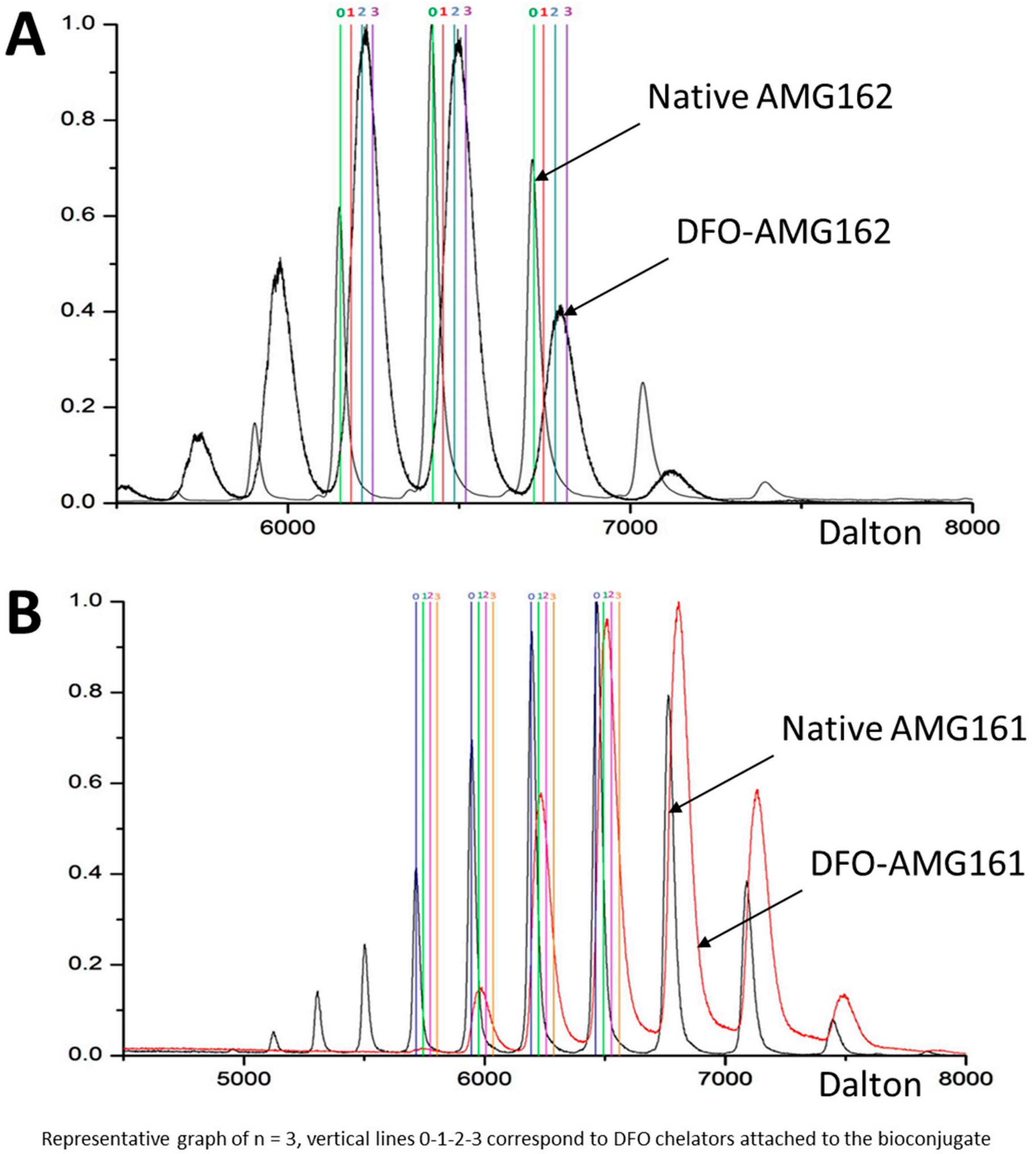

Figure S1. ESI-QTOF2 deconvoluted mass spectrometry data of bioconjugate A) DFO-AMG162 and B) DFO-AMG161 showing that bioconjugation yielded between 0 and 3 DFO chelators (abundant peak 2 DFO attached).

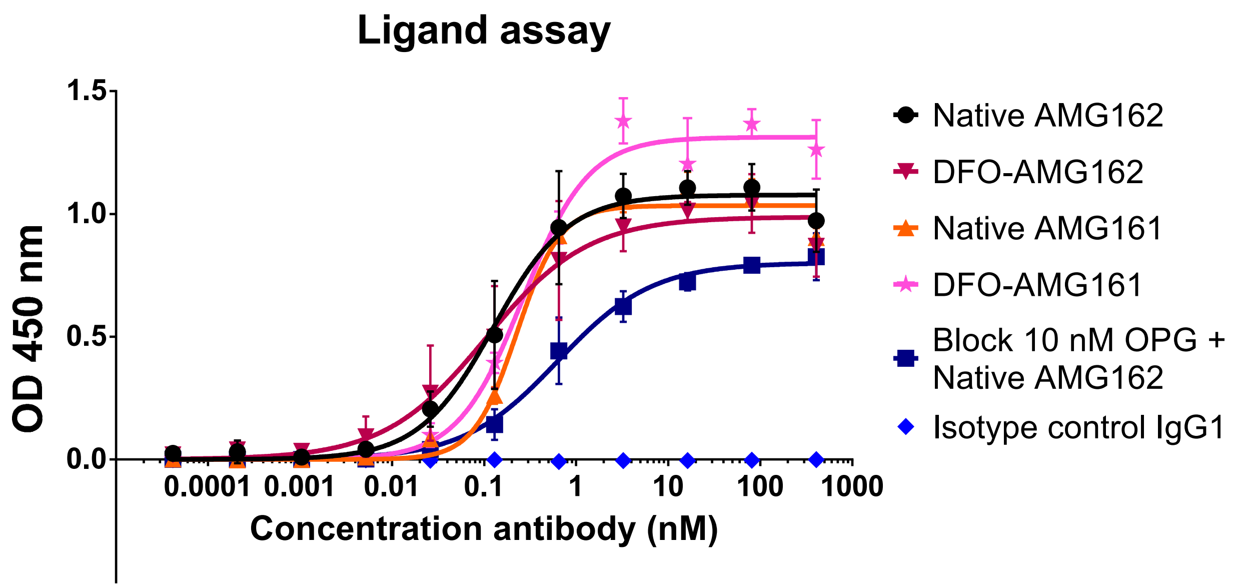

Figure S2. Binding affinity and specificity - Briefly, Immulon 2HB high-binding 96-well (Thermo Fisher Scientific, Waltham, MA, USA) plates were coated with 100 µL of a recombinant human RANKL solution (huRANKL; antigen; 10 nM; Amgen Inc., Thousand Oaks, CA, USA) at 4 °C overnight. Following this, the plates were washed three times with 0.2 % Tween/PBS, and then blocked with a 3 % bovine serum albumin (BSA)/PBS solution for 1 h at room temperature to avoid non-specific binding. The blocking solution was subsequently removed, and the wells washed three times with 0.2 % Tween/PBS. A dilution series was performed in triplicate using unmodified AMG161, AMG162, isotype control (IgG1, Amgen Inc., Thousand Oaks, CA, USA) and the DFO immunoconjugates. Samples were added to the first well (60 µg/mL, 125 µL) and 1/5 serial dilution was performed 10 times yielding a concentration range of 60 to 6 x 10-6 mg/mL. Binding specificity on native AMG162 as a blocking control was evaluated by using preincubation with OPG (10 nM; Cat# 450-14, Peprotech, London, UK). After 1 h incubation at room temperature, the antibody solutions were removed, and the wells washed three times with 0.2 % Tween/PBS. This was followed by the addition of rabbit anti-human horseradisch peroxidase (HRP) secondary antibodies (Abcam, London, UK, Cat# ab6759, RRID:AB_955434) (1/130,000 dilution, 2 mg/mL stock, 100 µL) and incubation for 1 h at room temperature. After washing three times with 0.2 % Tween/PBS, 100 µL HRP substrate (1:1 TMB substrate kit, 100 µL, Thermo Fisher Scientific, Waltham, MA, USA) was added to the wells and incubated for 20 min at room temperature. The color reaction was stopped by adding 100 µL 1 M H2SO4 (Sigma Aldrich, Saint Louis, MO, USA) stopping solution. The plates were read at 450 nm, and plotted data were analyzed to determine half-maximal effective concentration values using GraphPad.

Stability of radio-immunoconjugates in vitro

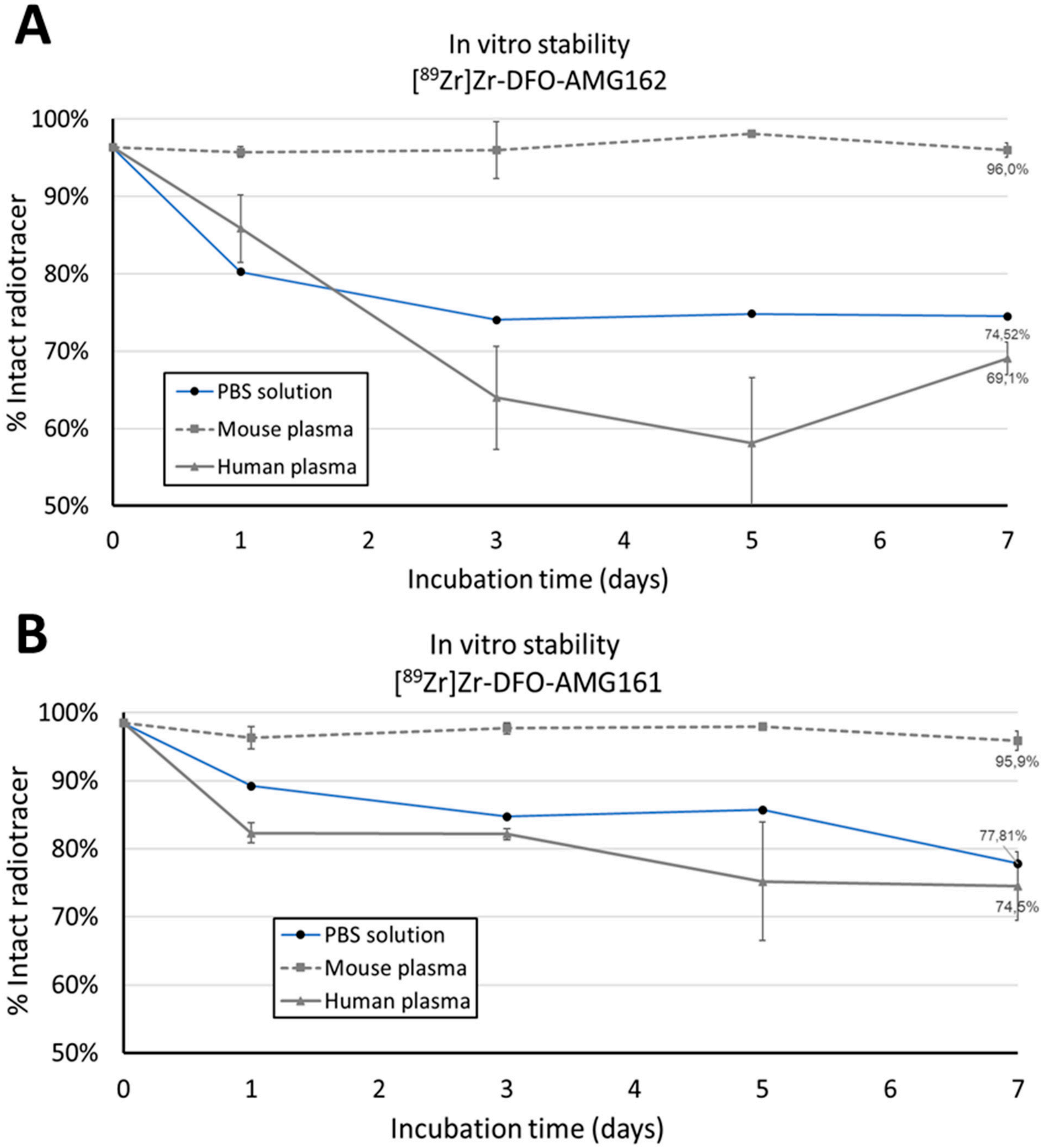

Figure S3. –Follow up stability in vitro of A) [^{89}Zr]Zr-DFO-AMG162 and B) [^{89}Zr]Zr-DFO-AMG161 in final formulation at room temperature, and in mouse and human plasma at 37°C, showed that the radiotracer remains stable in all tested conditions during at least 7 days. (n=3, data graph: mean +/- 1 standard deviation).

Biodistribution in Healthy CD-1 nude mice

Complete biodistribution and ex vivo analysis (%ID/g) was performed for [^{89}Zr]Zr-DFO-AMG162 and [^{89}Zr]Zr-DFO-AMG161 at 5 timepoints (day 1,2,3,4,7 post injection). Data is shown below as mean ± 1 standard deviation at each timepoint.

Table S1. – Ex vivo biodistribution (mean ± 1 standard deviation % ID/g) in healthy CD-1 nude mice (n = 4) at 1, 2, 3, 4, 7 days post injection of [^{89}Zr]Zr-DFO-AMG162.

**Organ**	Day 1 p.i.	Day 2 p.i.	Day 3 p.i.	Day 4 p.i.	Day 7 p.i.
Blood	15.08±3.9	13.33±0.7	12.34±2.0	12.93±1.5	9.42±1.2
Heart	3.66±0.7	3.68±0.6	3.21±0.4	3.15±0.2	2.68±0.4
Lungs	5.14±1.5	5.30±0.7	5.03±1.0	5.43±0.5	4.87±0.9
Liver	2.86±0.8	2.84±0.2	2.68±0.3	3.11±0.3	2.36±0.1
Spleen	2.98±1.2	3.18±0.3	3.33±0.7	3.88±0.4	3.77±1.0
Pancreas	1.27±0.2	1.18±0.1	1.19±0.2	1.25±0.1	1.05±0.2
Stomach	1.63±0.4	1.28±0.2	1.19±0.3	1.34±0.2	1.12±0.2
Small intestine	1.03±0.2	0.99±0.1	0.86±0.2	1.02±0.1	0.72±0.1
Large intestine	0.82±0.2	0.72±0.1	0.70±0.1	0.82±0.1	0.58±0.1
Kidneys	4.08±1.1	4.02±0.4	4.10±0.5	4.48±0.5	3.50±0.4
Bladder	4.59±0.5	4.43±0.3	4.23±0.8	4.55±0.3	3.81±0.7
Urine	0.83±0.2	0.58±0.1	0.63±0.3	0.46±0.2	0.41±0.1
Muscle	1.41±0.1	1.39±0.3	1.21±0.2	1.08±0.1	0.84±0.1
Fat	1.67±1.0	1.53±0.1	1.68±0.3	1.57±0.3	1.18±0.4
Bone	3.42±0.9	4.16±1.0	5.41±1.1	4.83±0.5	4.86±1.0
Brain	0.39±0.1	0.34±0.02	0.38±0.1	0.31±0.03	0.30±0.1
Skin	4.90±0.5	4.44±0.7	4.60±0.2	4.49±0.4	3.46±0.4

Table S2. – Ex vivo biodistribution (mean ± 1 standard deviation % ID/g) in healthy CD-1 nude mice (n = 4) at 1, 2, 3, 4, 7 days post injection of [^{89}Zr]Zr-DFO-AMG161.

**Organ**	Day 1 p.i.	Day 2 p.i.	Day 3 p.i.	Day 4 p.i.	Day 7 p.i.
Blood	6.83±5.2	7.31±4.0	2.76±3.3	2.98±2.2	2.39±1.5
Heart	2.64±0.8	2.41±1.0	1.49±0.5	1.51±0.5	1.33±0.4
Lungs	3.61±1.8	3.47±1.0	2.17±1.0	2.43±1.1	2.01±0.5
Liver	8.02±2.3	4.86±0.3	6.83±1.5	7.42±1.6	4.76±1.5
Spleen	12.09±5.8	4.71±0.7	8.04±4.6	12.08±4.2	8.14±3.0
Pancreas	1.15±0.1	1.14±0.1	0.81±0.3	0.87±0.1	0.80±0.1

Stomach	1.49±0.3	1.42±0.2	1.09±0.4	1.18±0.5	0.91±0.2
Small intestine	2.27±0.7	1.16±0.2	1.10±0.1	1.56±0.4	1.05±0.4
Large intestine	1.50±0.3	0.83±0.1	0.78±0.2	0.96±0.4	0.73±0.2
Kidneys	3.93±1.0	3.75±0.9	2.91±0.8	2.89±0.4	2.82±0.7
Bladder	3.65±0.8	3,57±0.6	2.54±1.2	2.67±0.8	2.23±0.1
Urine	1.08±0.6	0.91±0.4	0.76±0.1	0.69±0.04	0.39±0.1
Muscle	0.84±0.2	0.83±0.1	0.60±0.3	0.55±0.1	0.56±0.2
Fat	1.05±0.6	1.24±0.7	1.19±1.1	0.63±0.3	0.72±0.2
Bone	4.43±1.8	4.59±0.9	4.86±1.7	7.20±0.8	7.49±2.6
Brain	0.18±0.1	0.21±0.1	0.09±0.1	0.13±0.1	0.13±0.1
Skin	5.90±0.9	5.27±0.4	3.99±1.4	4.18±1.0	4.23±0.9

[89Zr]Zr-DFO-AMG162(IgG2) PET imaging in ME-180-RANKL xenografts

Table S3. – Ex vivo biodistribution (mean ± 1 standard deviation % ID/g) and tumor/organ ratio in healthy CD-1 nude mice (n = 5) at 5 days post injection of [89Zr]Zr-DFO-AMG162 in ME-180-RANKL xenografts.

	Mouse 1	Mouse 2	Mouse 3	Mouse 4	Mouse 5	Mean	SD
Tumor	24.13	29.99	26.10	28.82	21.91	26.19	3.32
Blood	5.49	7.32	6.33	5.05	5.32	5.90	0.93
Heart	1.76	2.02	1.53	1.86	1.47	1.73	0.23
Liver	3.28	3.50	3.76	4.58	2.92	3.61	0.63
Spleen	3.35	4.46	3.24	3.46	2.73	3.45	0.63
Kidneys	2.76	3.29	2.76	2.71	2.06	2.72	0.43
Muscle	0.54	0.80	0.66	0.60	0.47	0.61	0.13
Bone	5.69	7.69	7.71	7.57	6.90	7.11	0.86
Tumor/Blood	4.40	4.10	4.12	5.71	4.12	4.49	0.69
Tumor/Heart	13.73	14.86	17.07	15.51	14.90	15.21	1.22
Tumor/Liver	7.36	8.58	6.93	6.29	7.50	7.33	0.84
Tumor/Muscle	44.53	37.62	39.51	47.97	46.94	43.32	4.56
Tumor/Bone	4.24	3.90	3.39	3.81	3.18	3.70	0.42

Table S4. – Ex vivo biodistribution (mean ± 1 standard deviation % ID/g) and tumor/organ ratio in healthy CD-1 nude mice (n = 5) at 5 days post injection of [89Zr]Zr-DFO-AMG162 blocked ME-180-RANKL xenografts.

	Mouse 1	Mouse 2	Mouse 3	Mouse 4	Mouse 5	Mean	SD
Tumor	6.33	9.30	10.70	9.21	9.70	9.05	1.63
Blood	8.48	10.61	13.28	9.85	11.96	10.84	1.86
Heart	2.77	2.84	3.99	3.07	4.35	3.40	0.72
Liver	2.50	3.11	3.21	3.02	3.70	3.11	0.43
Spleen	3.72	5.21	6.22	5.90	5.89	5.39	1.00
Kidneys	3.66	3.67	4.02	3.02	4.73	3.82	0.62
Muscle	1.02	0.69	1.01	0.93	1.54	1.04	0.31
Bone	2.98	6.62	7.01	6.58	4.12	5.46	1.80

Tumor/Blood	0.75	0.88	0.81	0.93	0.81	0.83	0.07
Tumor/Heart	2.28	3.27	2.68	3.00	2.23	2.69	0.45
Tumor/Liver	2.54	3.00	3.33	3.05	2.62	2.91	0.33
Tumor/Muscle	6.22	13.42	10.58	9.90	6.30	9.28	3.06
Tumor/Bone	2.12	1.41	1.53	1.40	2.35	1.76	0.45

[^{89}Zr]Zr-DFO-AMG162 PET imaging in UM-SCC-22B/HCT-116 xenografts

Table S5. – Ex vivo biodistribution (mean ± 1 standard deviation % ID/g) and tumor/organ ratio in healthy CD-1 nude mice (n = 4) at 5 days post injection [^{89}Zr]Zr-DFO-AMG162 in UM-SCC-22B xenografts.

5 Days p.i.	Mouse 1	Mouse 2	Mouse 3	Mouse 4	Mean	SD
Tumor	6.36	6.74	6.16	6.79	6.51	0.31
Blood	9.78	8.97	9.98	15.94	11.17	3.21
Heart	2.79	2.70	2.57	3.99	3.01	0.66
Lungs	5.61	5.96	7.43	9.22	7.05	1.64
Liver	2.45	2.44	2.18	3.82	2.72	0.74
Spleen	3.60	3.73	3.69	5.78	4.20	1.05
Pancreas	0.89	0.86	0.73	1.47	0.99	0.33
Stomach	1.11	0.98	0.57	1.67	1.08	0.45
Small intestine	0.88	0.88	0.70	1.16	0.90	0.19
Large intestine	0.76	0.47	0,40	0,63	0,57	0,16
Kidneys	3.94	4.24	2.97	5.28	4.11	0.95
Bladder	3.26	4.03	3.39	4.64	3.83	0.63
Urine	0.32	1.06	0.28	0.52	0.54	0.36
Muscle	0.89	0.69	0.72	1.02	0.83	0.15
Fat	1.21	1.00	1.24	2.54	1.50	0.70
Bone	2.95	3.20	4.01	5.84	4.00	1.31
Brain	0.26	0.25	0.27	0.45	0.31	0.09
Skin	3.60	3.59	3.87	5.53	4.15	0.93
Tumor/Blood	0,65	0,75	0,62	0,43	0,61	0,14
Tumor/Heart	2,28	2,50	2,40	1,70	2,22	0,35
Tumor/Liver	2,59	2,77	2,83	1,78	2,49	0,49
Tumor/Muscle	7,15	9,78	8,56	6,68	8,04	1,41
Tumor/Bone	2,16	2,11	1,54	1,16	1,74	0,48

Table S6. – Ex vivo biodistribution (mean ± 1 standard deviation % ID/g) and tumor/organ ratio in healthy CD-1 nude mice (n = 5) at 5 days post injection [^{89}Zr]Zr-DFO-IgG2 in UM-SCC-22B xenografts.

5 Days p.i.	Mouse 1	Mouse 2	Mouse 3	Mouse 4	Mouse 5	Mean	SD
Tumor	4.92	5.56	6.31	4.31	5.90	5.40	0.79
Blood	10.84	15.54	14.43	13.58	12.12	13.30	1.86
Heart	3.10	3.32	4.05	2.94	3.21	3.32	0.43

Lungs	6.00	5.39	6.35	5.56	3.96	5.45	0.92
Liver	3.00	3.71	3.69	3.31	3.77	3.50	0.33
Spleen	4.87	5.59	5.11	4.61	5.17	5.07	0.36
Pancreas	0.73	1.21	0.94	0.99	0.98	0.97	0.17
Stomach	0.72	1.20	0.94	1.16	0.94	0.99	0.20
Small intestine	0.73	0.81	0.48	0.75	0.64	0.68	0.13
Large intestine	0.59	0.63	0.67	0.50	0.54	0.59	0.07
Kidneys	3.29	3.83	3.66	3.22	3.31	3.46	0.27
Bladder	3.63	4.02	3.87	3.80	3.05	3.68	0.38
Urine	0.45	0.54	0.30	0.57	0.57	0.49	0.12
Muscle	0.58	0.93	0.89	0.73	0.72	0.77	0.14
Fat	2.37	1.44	1.86	2.10	1.03	1.76	0.53
Bone	2.25	4.87	2.19	3.58	1.50	2.88	1.35
Brain	0.20	0.23	0.29	0.24	0.25	0.24	0.03
Skin	2.83	4.38	4.03	4.71	2.94	3.78	0.85
Tumor/Blood	0.45	0.36	0.44	0.32	0.49	0.41	0.07
Tumor/Heart	1.59	1.68	1.56	1.46	1.84	1.62	0.14
Tumor/Liver	1.64	1.50	1.71	1.30	1.56	1.54	0.16
Tumor/Muscle	8.47	5.95	7.12	5.89	8.17	7.12	1.20
Tumor/Bone	2.19	1.14	2.89	1.20	3.92	2.27	1.18

Table S7. – Ex vivo biodistribution (mean ± 1 standard deviation % ID/g) and tumor/organ ratio in healthy CD-1 nude mice (n = 5) at 5 days post injection [^{89}Zr]Zr-DFO-AMG162 in HCT-116 xenografts.

5 Days p.i.	Mouse 1	Mouse 2	Mouse 3	Mouse 4	Mouse 5	Mean	SD
Tumor	5.48	5.86	5.87	5.63	4.70	5.51	0.48
Blood	11.37	12.24	11.87	13.42	10.80	11.94	0.99
Heart	2.45	3.18	2.79	3.23	2.89	2.91	0.32
Lungs	6.42	5.45	8.45	5.87	5.64	6.37	1.22
Liver	3.29	2.74	2.76	2.70	2.78	2.85	0.25
Spleen	5.13	5.65	5.07	6.10	5.22	5.44	0.44
Pancreas	1.11	1.10	1.24	1.12	0.94	1.10	0.11
Stomach	1.04	0.81	1.18	0.98	1.07	1.02	0.14
Small intestine	1.08	0.87	0.87	1.03	0.85	0.94	0.11
Large intestine	0.89	0.78	0.71	0.82	0.56	0.75	0.12

Kidneys	4.22	4.33	4.08	3.91	3.68	4.04	0.26
Bladder	4.20	3.87	4.15	4.75	3.82	4.16	0.37
Urine	0.47	0.61	0.51	0.52	0.58	0.54	0.06
Muscle	1.00	0.68	0.84	0.86	0.93	0.86	0.12
Fat	2.87	1.70	1.88	2.29	1.43	2.03	0.56
Bone	5.50	3.63	4.16	3.19	3.61	4.02	0.90
Brain	0.31	0.29	0.24	0.31	0.29	0.29	0.03
Skin	3.71	4.23	3.78	4.44	3.39	3.91	0.42
Tumor/Blood	0.48	0.48	0.49	0.42	0.43	0.46	0.03
Tumor/Heart	2.24	1.84	2.10	1.74	1.62	1.91	0.26
Tumor/Liver	1.67	2.14	2.12	2.08	1.69	1.94	0.24
Tumor/Muscle	5.49	8.62	6.96	6.52	5.05	6.53	1.40
Tumor/Bone	1.00	1.61	1.41	1.77	1.30	1.42	0.30

IHC quantification

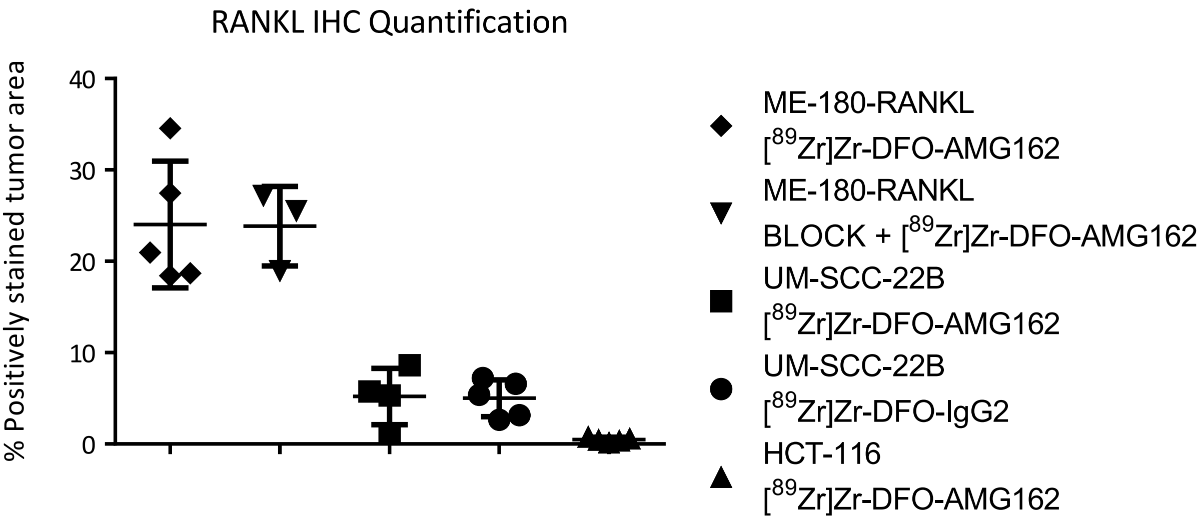

Figure S4. – RANKL IHC quantification (DAB brown stain) in slides of different evaluated xenografts ME-180-RANKL ([^{89}Zr]Zr-DFO-AMG162 (n = 5)), ME-180-RANKL + Blocking ([^{89}Zr]Zr-DFO-AMG162 (n = 3)), UM-SCC-22B ([^{89}Zr]Zr-DFO-AMG162 (n = 4) + [^{89}Zr]Zr-DFO-IgG2 (n = 5)) and HCT-116 ([^{89}Zr]Zr-DFO-AMG162 (n = 5)) Briefly, the sections were fixed in 4 % paraformaldehyde (Sigma Aldrich, Saint Louis, MO, USA), rehydrated (x6) in PBS and incubated for 3 min with peroxidase block (DAKO). After washing 3 x 5 min in PBS, sections were blocked with PBS/triton-X100 5 % normal goat serum and incubated for 1 h at room temperature. Primary antibody incubation (anti-huRANKL; (1:500) dilution, Thermo Fisher Scientific, Waltham, MA, USA, Cat# PA5-21951, RRID:AB_11156181) was performed overnight at 4 °C. Secondary antibody incubations and DAB were performed according to the manufacturer's instructions (DAKO). The nuclei of the tumor sections were counterstained using Mayer hematoxylin (Sigma Aldrich, Saint Louis, MO, USA). Appropriate positive (human lymph node tissue) and negative control samples (mouse lymph node, brain tissue) were included and confirmed the absence of cross-reactivity between human and mouse RANKL. Light microscopic images were captured using an Eclipse Ti microscope (Nikon, Melville, NY, USA) (internal magnification factor ×10) with ×4 (overview images) and ×20 (detail images) objectives.
